# Spatial and temporal characterization of *Aedes albopictus* oviposition activity in candidate urban settings for sterile insect technique testing in La Reunion Island

**DOI:** 10.1186/s40249-024-01250-z

**Published:** 2024-10-25

**Authors:** Gilbert Le Goff, David Damiens, Abdoul-Hamid Ruttee, Frédéric Jean, Laurent Payet, Cyrille Lebon, Paul Taconet, Benjamin Gaudillat, Nausicaa Habchi-Hanriot, Jean-Sébastien Dehecq, Frédéric Simard, Louis-Clément Gouagna

**Affiliations:** 1 Unité Mixte de Recherche “Maladies Infectieuses et Vecteurs: Écologie, Génétique, Évolution et Contrôle” (MIVEGEC): Université Montpellier, Institut de Recherche pour le Développement, Centre National de Recherche Scientifique, Délégation Régionale Occitanie, Montpellier, France; 2 Institut de Recherche pour le Développement (IRD) La Réunion/Groupement d’Intérêt Public (GIP) Cyclotron Océan Indien, Recherche Santé Bio-Innovation, Sainte Clotilde, Reunion Island France; 3 Service de lutte anti vectorielle, Agence Régionale de Santé-Océan Indien (ARS-OI), Saint-Denis, Reunion Island France

**Keywords:** Urban environment, *Aedes albopictus*, Ovitrap, Egg abundance, Spatiotemporal variability

## Abstract

**Background:**

Understanding of mosquito spatiotemporal dynamics is central to characterize candidate field sites for the sterile insect technique (SIT) testing, and is critical to the effective implementation and evaluation of pilot sterile male release programs. Here, we present a detailed description of *Aedes albopictus* (Skuse) egg-laying activity over a 6-year period in urban areas identified as potential SIT testing sites on Reunion Island.

**Method:**

Weekly entomological collections using ovitraps were carried out in residential and adjacent uninhabited habitats in two urban areas, Duparc and Bois Rouge, in the municipality of Sainte Marie, Reunion Island. Time-series data incorporating the frequency of positive ovitraps and the total number of eggs/ovitrap recorded each time at each locality during the study period from May 2013 to December 2018 were analyzed with multifaceted statistical approaches including descriptive statistics and spatiotemporal analyses incorporating the role of climatic factors on overall ovitrap productivity.

**Results:**

During the ovitrap survey, the proportion of egg-positive ovitraps differed among study sites (*χ*^*2*^ = 50.21, df = 2, *P* < 0.001), being relatively lower in Duparc (89.5%) than in Bois-Rouges (95.3%) and the adjacent buffer zone (91.2%). Within each neighborhood, *Ae. albopictus* egg abundance varied by month in a roughly seasonal pattern marked by a single peak occurring more regularly February each year, a decline at the onset of the austral winter in July, followed by a period of lower ovitrap productivity in August and September. Fluctuation in both positivity rate and eggs densities per ovitraps were related to annual and seasonal variations in local temperature and rainfall (*P* < 0.001 in all cases). The spatial analysis also captured substantial between- and within-habitats heterogeneity, whereby the overall ovitrap productivity was higher in residential areas than in the buffer zone.

**Conclusions:**

Collectively, these results reveal that the distribution of *Ae. albopictus* oviposition activity is shaped by local habitat heterogeneity and seasonal climatic factors. Overall, this study provides baseline insights into the reproductive dynamics of *Ae. albopictus*, which would assist in planning locally tailored SIT interventions, while addressing concerns related to focal areas of high egg-laying intensity and potential immigration of females from natural areas.

**Supplementary Information:**

The online version contains supplementary material available at 10.1186/s40249-024-01250-z.

## Background

Mosquito species diversity in Reunion Island includes 13 endemic species, of which *Culex quinquefasciatus*, *Anopheles arabiensis*, *Aedes aegypti,* and *Ae. albopictus* are known for their medical importance [1]. Among these, *Ae. albopictus* is by far the dominant species, occurring mainly over large geographical areas in urban environments. Several studies have been published that attempt to describe the potential geographic range of *Ae. albopictus* in Reunion Island and the South West of the Indian Ocean [2–4]. With a significant tourist industry, Reunion is concerned about the presence of *Ae. albopictus* and its widespread geographic distribution and abundant population. Favorable climatic conditions, including temperature and rainfall, contribute to the reproductive success and proliferation of this mosquito species in urban environments [5], while its anthropophilic behavior facilitated it being the key potential vector of arboviruses [4]. Vazeille et al. have experimentally demonstrated that *Ae. albopictus* from Reunion Island is an efficient alphavirus and flavivirus vector [6]. This mosquito species was causally responsible for the spread of the devastating chikungunya outbreak in 2006 [7] and the multiple epidemic waves of all four serotypes of dengue virus involving hundreds to thousands of cases, which have occurred every year on the island since 2014 [8]. Total costs of the Chikungunya outbreak in Reunion, including vector management costs and losses, were estimated at USD 365 million. Personal control measures associated with repellents, and insecticide treatment incurred by local populations add another USD 38 million annually due to high nuisance [9]. Dengue circulation now persists in an endemic state with intrinsic seasonality in transmission intensity. The progress into endemic status is now considered a new challenge creating the need to continuously manage the main vector to mitigate further outbreaks and re-emerging vector-borne diseases.

The increasing public health importance of dengue, the failure of conventional approaches to control the vector involved, and sustained expression of public concern regarding the unwanted side effects of insecticide spraying on non-target organisms and the potential evolution of resistance of *Ae. albopictus* to deltamethrin [10, 11], highlighted the need for more efficient and ecological alternatives. This situation has stimulated interest in innovative vector control strategies for improved prevention of vector-borne diseases. Among the known innovative strategies [12, 13], much attention has been brought to the use of the sterile insect technique (SIT), whereby radiation-sterilized males are mass-released in the wild for mating with wild females resulting in non-viable eggs and reduced wild populations in the subsequent generations [14, 15]. Historically, SIT has been designed and applied as a biological control method for pest eradication in agriculture [16]. For example, this technique has proven its effectiveness on the Mediterranean fruit fly (Medfly: *Ceratitis capitata*) and on New World screwworm (*Cochliomyia hominivorax*) [17]. This technique is currently being developed as an essential component of the strategies for the suppression, containment, prevention, and, if necessary, eradication of the vector species of human diseases [18, 19]. The approach has the advantage of being species-specific and has therefore no effect on non-target organisms.

Given the limitations of the current management strategy used against *Ae. albopictus*, a research program sponsored by the Ministry of Health and the Regional Council of Reunion was initiated in 2009 to develop and assess the technical, social, and economic feasibility of an area-wide SIT for targeted and long-term suppression of *Ae. albopictus* on the island [20]. Sterile male releases aim to control the fertility in wild females, which may ultimately translate into the reduction of adult population abundance over successive generations. The formulation of release strategies, as well as their further efficacy evaluation, are underpinned by a thorough understanding of the ecological processes involved in key life stages of the target population. Indeed, the response of a vector population to SIT depends on the initial level of population density or fecundity which reflects the activity of biting females [21], and its interactions with complex environmental factors. The greatest response to SIT will be expected at the time of year when the density of the field populations is the lowest or at least in populations already declining in fecundity and abundance.

The ecology of *Ae. albopictus* in geographically isolated areas such as La Reunion Island [22, 23] makes it a promising target for control by SIT. To prepare a SIT field testing against *Ae. albopictus*, different aim-oriented entomological studies, such as evaluation of temporal patterns of population densities using mark-release-recapture experiments and routine population monitoring using different trapping systems (e.g. [24, 25]), have been carried at selected urban environments to gather baseline entomological information for developing specific scheduled sterile male releases. Besides repeated measurements of adult egg-laying activity and egg fertility of the target species over a sufficient period of time are required to reflect mosquito population dynamics and understand the determinants of their variation. This information is crucial not only to estimate the timing of the release with effective use of labor and other expensive resources but also to inform SIT intervention strategy on how best to reduce the wild population. Here, we examined the temporal patterns as well as the spatial distribution of *Ae. albopictus* oviposition activity and their environmental determinants over two selected candidate urban areas in Reunion Island. Results from this study may help creating a comprehensive baseline entomological insight into the reproductive dynamic of *Ae. albopictus* [26], with practical implications for the implementation and evaluation of locally adapted SIT interventions over representative urban areas in Reunion Island.

## Methods

### Study sites

**Fig. 1:**
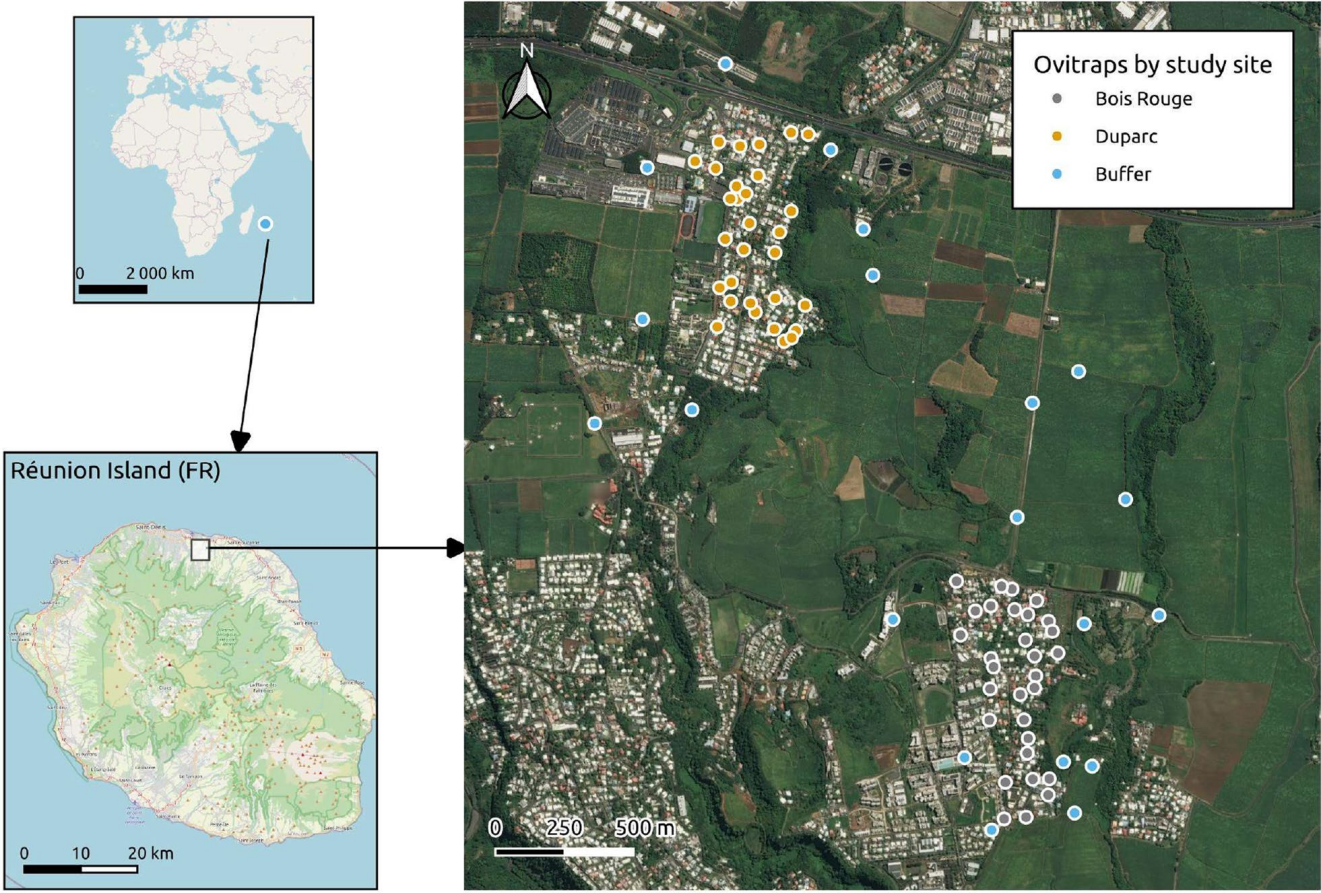
Sampling sites. Map showing the location of the study sites. Background layer: OpenStreetMap, Bing Aerial. Reunion Island (bottom left map) lies in the southern Indian Ocean, to the southeast of the African continent at a distance of 679 km east-southeast of Madagascar (upper left map). Each dot in the main map corresponds to the ovitrap positions and is colour-coded according to specific habitats defined by Duparc (yellow dots) and Bois Rouge (grey dots) and the buffer zone (blue dots)

In 2013, following discussions with the Vector Control Agency and positive feedback from the municipal council of Sainte Marie, two study sites were selected in an effort to establish the feasibility study of SIT for mosquito control. Sainte Marie (Latitude: 20° 52′ 60'' South, Longitude: 55° 32′ 58.6'' East) is a coastal municipality located 9 km southeast of Saint-Denis- Reunion Island, covering 87.2 km^2^ with a population of 34,344 since the last census in 2021**.** Priority was given to urbanized settlements where *Ae. albopictus* is the only *Stegomyia* species present. Other considerations taken into account for selecting the study sites were (a) areas with manageable sizes (range: 20–40 hectares) and well-delimited residential areas geographically isolated by natural barriers, (b) proximity to laboratory facilities, and accessibility by car. Accordingly, Duparc and Bois-Rouge, two urban areas within the municipality of Sainte Marie were selected (Fig. 1).

Duparc covers an area of approximately 42 ha at an altitude between 50 and 100 m above sea level (asl). With approximately 1885 inhabitants, it is a dense and homogeneous urban habitat with more than 94% of the land area almost exclusively made up of individual properties (about 300–500 households) with private gardens. The surrounding area features a variety of amenities and infrastructures for transport, education, healthcare services, business and shopping centers. The site is isolated by an expressway linking Saint-Denis to Saint Benoit to the north, by the rain-fed Ravine *La Mare*, and to the east, west, and south by sugar cane fields. The rain-fed ravine is a semi-natural wooded area made up of difficult to access landscape with rocks. The residential area is surrounded by sugarcane fields, except for the grassland located to the north, towards the sea.

The second site is Bois-Rouge, an urban area of about 24 ha extending over an elevation of approximately 150–200 m asl with an estimated population of 1745 inhabitants. The residential area is made up of slightly more heterogeneous individual housing spread over 21% of its total surface area (63% of the properties are individual houses and 37% are apartments) and interspersed with uninhabited land with few small poultry or goat farms. The neighborhood's most common socio-professional category is working-class people and farmers. The eastern and southern periphery of Bois-Rouge is mainly agricultural. A small, shallow ravine runs through the residential area, which is surrounded by market gardening fields, while the northwest part is bordered by sugarcane fields up to Duparc. A map showing ovitrap location in and around each site is presented in Fig. 1. Duparc forms the core for entomological studies to guide the implementation of the SIT- pilot trial for the suppression of *Ae. albopictus*, while Bois-Rouge serves as our control.

Lastly, a “buffer zone” was also defined as non-residential urban environments separating or surrounding the residential areas in Bois Rouge and Duparc. In brief, the buffer zone comprises an extended area of approximately 1 km separating Duparc and Bois-Rouge, in additional to the roughly 200 m zone bordering each urban site (Fig. 1). These landscapes are made up of open vegetation, sparse shrubs and grasses in parts, and agricultural fields that include vegetable gardens and sugarcane plantations. However, no specific of systematic habitat characteristics (such as land cover diversity, variability in vegetation, any topography data) were collected at the two neighborhoods or in the buffer strip around each site perimeter.

### Climate conditions

The climate in the study areas is typical oceanic tropical characterized by low annual, seasonal, and daily temperature variations. The climatic regime alternates between two seasons: the cool and rather dry season during the austral winter from June to October, and the rainy and warmer season from November to May, during which vast areas are directly affected by tropical depression-type phenomena or cyclones [27, 28]. La Reunion is regularly exposed to cyclonic events (tropical storms or cyclones), which generally occur between December and April, with strong rainfall and high wind speeds during January, February, and March. Supplementary **Table S1** shows the monthly weather parameters reported by the nearest “Gillot" weather station to our study sites, located at the Roland Garros international airport. Total annual precipitation averages 1530 mm, mostly spread over 6 months from December to May [29]. The average daily temperature is 22.5 °C, with a minimum below 19 °C in the coolest and driest months in July and August and a maximum of approximately 30 °C in January–February**.**

#### Longitudinal survey of *Ae. albopictus* oviposition activity

During the initial phase of the study, proactive dialogues with the residents were conducted to seek their approval before designing the mosquito sampling described herein. They were contacted individually during home visits and an information note explaining the initial project objective and summarizing the objectives of the entomological monitoring with trapping methods used was distributed to them to obtain their consent to enter their private property. No specific interventions for vector control were applied for both field sites during the study period. The fieldwork has been spread over the period from May 2013 and December 2018. The sampling strategy using oviposition traps (Ovitrap) was biased towards residential areas (Duparc and Bois Rouge) previously selected for SIT pilot testing. Except for 2016 (sampling in January and December) and 2017 (sampling in October, November, and December), each urban neighborhood was surveyed consistently in 2013, 2014, 2015, and 2018. The sampling strategies was extended between April 2014 and January 2016 to include non-residential locations, i.e. the buffer zone, mainly to examine whether natural and agricultural landscape surrounding the residential neighborhoods may represent favorable environments for *Ae. albopictus*. On each occasion, a total of 29, 27, and 20 ovitraps were operated in Bois-Rouge, Duparc, and the buffer zone separating the two urban areas, respectively (Fig. 1). Each ovitrap consisted of a 1.2-L black plastic pot filled with 650 ml of water collected from a river. The oviposition substrate (ovistrip) was made of a brown germination paper (400HPT Sedburo Equipment, Des Plaines, USA) or white filter paper (145 g/m^2^ Sartorius, Germany) (measuring 15× 10 cm) attached with a paper clip on a 15 × 10 cm smooth and rigid PVC sheet, which was placed inside the ovitrap. The papers were labeled on the back with a unique number, followed by the number of the week of collection. In each sampling zone, the ovitraps were usually placed at ground level in a shaded location or low vegetation and preferably sheltered from the wind and the sun, most frequently in volunteer’s private gardens and some were placed in the public domains and natural areas within and outside (buffer zone) of the residential neighborhoods. Each ovitrap had a unique identifier and GPS coordinates were recorded for each trap.

Quite remarkably, the ovitraps were very rarely overturned, damaged, or even lost (< 0.8% of ovitraps over the studied fields and the duration of the study), as all the traps deployed in the field were very well protected from potential disturbances. They were checked weekly during which egg-laying papers were collected and annotated with the trap number and collection date before being taken to the lab for egg counting. Any larva observed in the traps was recorded and identified by stage of development. The pots were completely emptied of their residual water and cleaned to remove any eggs that may have been deposited. They were filled with water before being set again at the exact same position, with a new substrate. In the laboratory, filter papers were cleaned and checked for the presence of mosquito eggs. Eggs were counted either immediately or after drying and maturation of the embryos when field-collected egg samples were used to evaluate the hatching rate. In the first case, the number of eggs from each weekly sample was recorded, based on three scores: i) the overall number of eggs observed on each ovitrap substrate, ii) the number of opened eggs when the opercula are separated from the rest of the eggshell (corresponding to viable eggs); iii) and the number of eggs that have a completely flattened appearance (corresponding either to non-embryonated eggs or to non-viable embryos). The weekly, monthly, and annual average number of eggs/trap were calculated and used as a proxy of *Ae. albopictus* abundance in each study site. It is worth noting that we never observed eggs of other mosquito species in the ovitraps during the study, and all eggs recovered in the field were identified as those of *Ae. albopictus.* The viability of *Ae. albopictus* eggs collected in the field was assessed every other week in Duparc (the priority zone for future SIT testing) and every month in Bois-Rouge in 2013–2014, by comparing the number of open versus closed eggs out of the total number of eggs in a positive trap collection, while controlling for external weather conditions. Verification of the egg viability was discontinued from 2015 onwards because the overall hatching rates of eggs collected in individual habitats on any one week in 2013–2014 consistently reached or even exceeded 90%, regardless of the location and the season. Although it would have been desirable to record the egg-hatch rate for all the samples collected each year, it was not feasible to work with larger samples.

### Meteorological data

Records of the daily rainfall (in mm), minimum, maximum, and mean temperature (in degrees Celsius), average daily temperature amplitude (°C), daily minimum, maximum, and mean relative humidity (%), as well as average daily wind velocity (m/s), total daily sunshine duration (mn), and cumulative daily global solar radiation (J/cm^2^) in Sainte Marie district were obtained from the Gillot Airport meteorological station [29], located within 1 km and 2 km distance of Duparc and Bois-Rouge, respectively. These factors were considered because they are known to better explain differences between populations of our target species [30]. The climatic data were aggregated to provide monthly estimates for each variable (Supplementary file 1). As the two study areas of Duparc and Bois-Rouge are located 1 km apart, they are considered identical, so spatial heterogeneity in these weather parameters is negligible**.**

### Statistical analysis

The study period covered data from 2013 to 2018. Inevitably, the sampling was partially interrupted on some occasions due to unfavorable weather, in which case monitoring had to stop until it was possible to continue again. In practice, field activities were particularly difficult during the cyclone. Any such interruption was recorded as 'time out'. Despite this, the period covered by this report encompasses a period when the sampling was conveniently made and valid data recorded (Supplementary file 1). The final dataset comprising the number of positive ovitraps and number of eggs collected per ovitrap at each location is a combination of observations made at different time periods. Specifically, data for the urban study sites of Duparc and Bois-Rouge span from May 2013 to January 2016, December 2016, October–November 2017 and January–December 2018, while data for the buffer zone cover a period from April 2014 to January 2016 on a weekly basis. The resulting records include over 10,143 (Duparc: 4569; Bois-Rouge: 4626; buffer zone: 948) weekly ovitraps records over the whole study period. Variation in the number of observations between locations arose from the irregular or inconsistent monitoring over time. For the sake of comparability between study sites and to account for variations in the length of the sampling periods within each location [31], thereby minimizing variability resulting from missing data (see [32]), all weekly data for a given site are reported as the sum and average of all data over the sampling months and years, split by seasons.

We first examined the temporal variation of the proportion of ovitraps that were egg-positive and the egg abundance over the entire sampling period across studied sites. Subsequently, monthly data were standardized by grouping into wet season: wet and hot summer (November–May) and warm-dry winter (June–October) to allow comparison within and among areas of interest, both in terms of the presence/absence and the abundance of eggs across seasons. We successively investigated the relationship between the dependent variables (number of egg-positive ovitraps treated as proportion and actual egg counts) and several potential covariates by fitting a Generalized Linear Mixed Model (GLMM) using a negative binomial link function with gamma distribution in SPSS package for Windows (IBM SPSS Statistics 22.1, Armonk, USA). The explanatory variables considered in the GLMM were habitat type (a categorical variable with two levels: residential, non-residential), season (categorical with two levels: dry and rainy) and all the 11 continuous variables representing weather parameters obtained from nearby meteorological station. These covariates were included as fixed effects, while a random intercepts were added for the study locations (categorical with three levels: Duparc Bois Rouge and Buffer zone), and the ovitraps collection points as random effect, accounting for repeated measures in the same sites and at the same sampling location. A theoretical approach based on the Akaike Information Criteria (AIC) was used to select the most plausible statistical models that satisfactorily explain the egg abundance observations. The best models were considered to be those associated with the smallest AICc, and a value-of-evidence threshold of ΔAICc ≤ 2 was adopted to select the most plausible models [33]. For the models selected as best we verified the normality, homoscedasticity (i.e., equal variance) and independence assumptions of the error [34]. In addition, generalized linear models (GLM) with Poisson distribution were carried out on ovitrap data to assess whether the prevalence of eggs positive ovitraps and the egg density/ovitrap differed between habitats, month, and/or year season. All continuous variables were log-transformed prior to analyses. Study site was included as additional, fixed explanatory variable in all analyses so that the effect of time could be evaluated while controlling for between-habitat variation in ovitrap productivity. By including monthly records as data points, we were able to incorporate season (summer, winter) as a potentially critical explanatory variable into our statistical analysis. As climatic factors are important determinant of behavior, and in particular oviposition, it was essential to take these parameters into account when testing for any effect of season. The maximal statistical model included all explanatory variables and their group interactions. All non-significant terms were sequentially dropped to yield a minimum model. The test statistic for all general linear models analyses was the F-value, and the significance level (α) for all statistical tests was set at 0.05. Following a significant test, Furthermore, for this analysis, the Bonferroni correction was applied to counteract the problem of multiple comparisons and avoid type I errors [35]. The relationships between monthly population fluctuations of mosquitoes with meteorological variables were further evaluated by Pearson’s correlation coefficient.

In the spatial analysis we assumed the ovitraps were independent – being placed in different positions within the study areas, therefore, we used the GIS-based Multi Criteria Evaluation (MCE) approach to produce maps to determine if there is high heterogeneity in egg abundance at the landscape scale in Bois-Rouge and Duparc. Kriging interpolation was used to estimate the seasonal abundance of *Ae. albopictus* at unsampled locations using data obtained by fixed ovitrap sampling throughout each study area. In addition, cluster analysis was used to identify the level of expected between-location clustering of particular areas that had consistently significant higher or lower egg density. Determination of the level of expected between-location clustering may provide information on environmental variables that determine species diffusion and that should be considered in guiding SIT intervention and evaluation. All data were statistically analyzed using SPSS statistical package for Windows (version 22.1, Armonk, NY: IBM Corp. 2020) and in R Statistical Software (v3.6.2; R Core Team 2019, Vienna, Austria). Spatial analysis examining the properties of seasonal and spatial patterns and trends of *Ae. albopictus* abundance was performed using SatScan™, while specific geographic representations of the distribution of egg abundance and density were drawn using the R software (v.3.6.2) with the ‘ggplot2’, ‘ggmap’ and ‘ggdensity’ packages [36, 37].

## Results

### Variation in the proportion of egg-positive ovitraps by season and habitat type

**Fig. 2:**
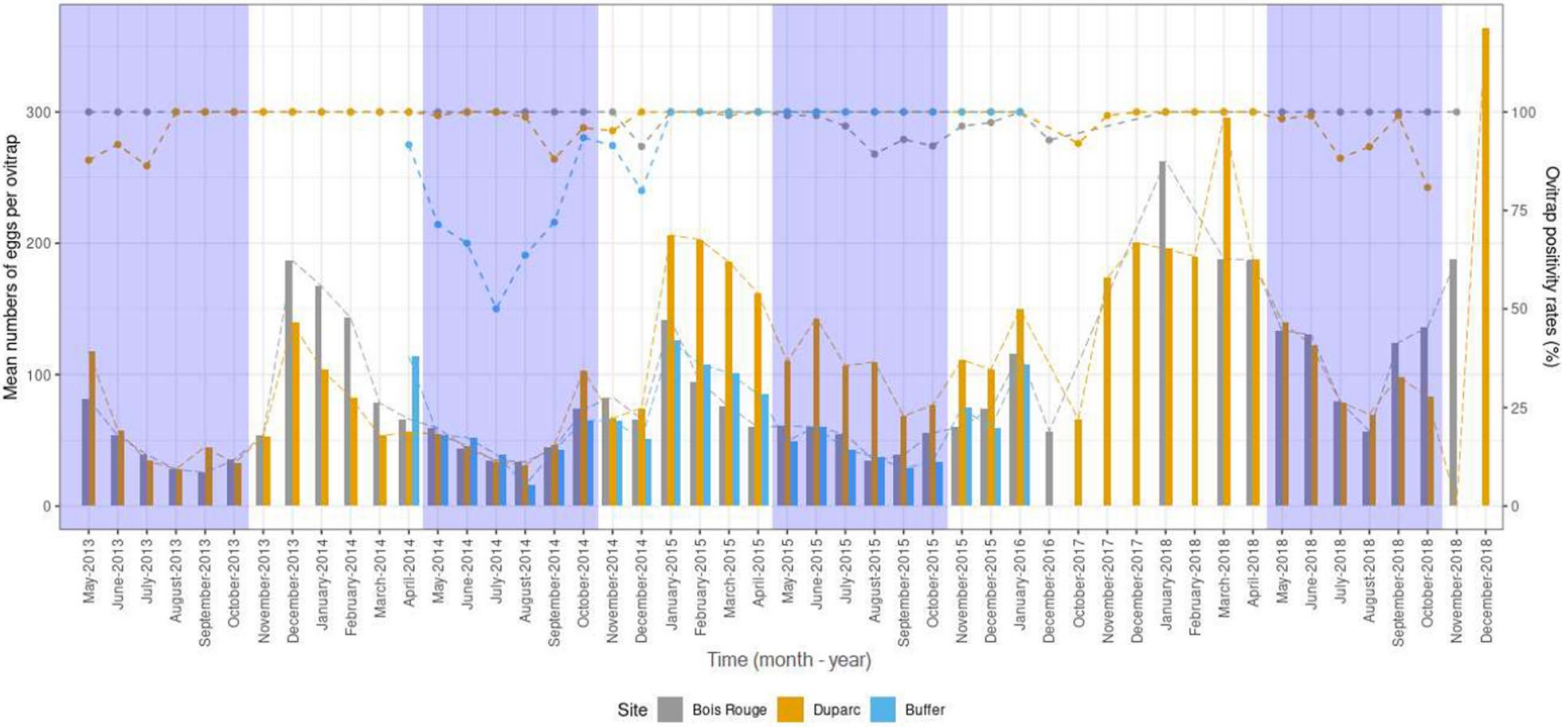
Time series of monthly records of ovitrap positivity rates (%) and average numbers of *Ae. albopictus* eggs per ovitrap through the study period (May 2013 to December 2018). Dots represent average proportions of egg-positive ovitraps, and bars represent a time series of the average number of eggs recorded per ovitrap at each sampling month. The dry winter season (May–October) is shaded light grey

The time interval covering the survey was from week 20 in 2013 to week 52 in 2018, resulting in a total of 204 weeks of monitoring using 26.6 (± 0.8) and 28.6 (± 0.5) ovitraps in Duparc and Bois-Rouge, respectively. For the timeframe considered in this study the ovitrap positivity (i.e. proportion of egg-positive ovitraps over the total number of traps in the studies areas) varied between habitat type (Likelihood Ratio Chi-square, *χ*^*2*^ = 50.21, df = 2, *P* < 0.001), being relatively lower in Duparc (89.5%, 4090/4569) than in Bois-Rouges (95.3%, 4408/4626) and the buffer zone (91.2%, 865/948). Overall, the proportion of positive ovitrap was much greater if ovitraps were placed in the residential area compared to the adjacent buffer zone (residential vs buffer zone: *χ*^*2*^ = 38.34, df = 1, *P* < 0.001). There were significant variations in the proportion of egg-positive traps between the sampling years in Duparc (*χ*^*2*^ = 79.77, df = 5, *P* < 0.001), Bois-Rouge (*χ*^*2*^ = 83.94, df = 4, *P* < 0.001), and in the buffer zone (*χ*^*2*^ = 123.17, df = 2, *P* < 0.001). Regardless of the habitats where ovitraps had been set, both in the residential areas in Duparc and Bois-Rouge, the proportion of ovitraps producing eggs tended to vary between months each year (*χ*^*2*^ = 25.79, df = 11, *P* = 0.007). As shown in Fig. 2 the seasonal pattern of the percentage of egg-positive ovitrap was similar in all study sites, being higher in the summer (nearly 100%) compared to the winter. In each studied location, the proportion of egg-positive ovitraps was constantly at its highest (99–100%) in November and April, and declined between May and October (Fig. 2), reflecting a possible decrease in the activity of *Ae. albopictus* females following the onset of the austral winter. Comprehensively, the lowest positivity rates were recorded in this season at Duparc during May–July 2013 and September 2018 with 88% egg-positive ovitraps, at Bois-Rouge with 91% recorded from August to September 2015 and in the buffer zone with only 56% observed in July and August 2014.

**Fig. 3:**
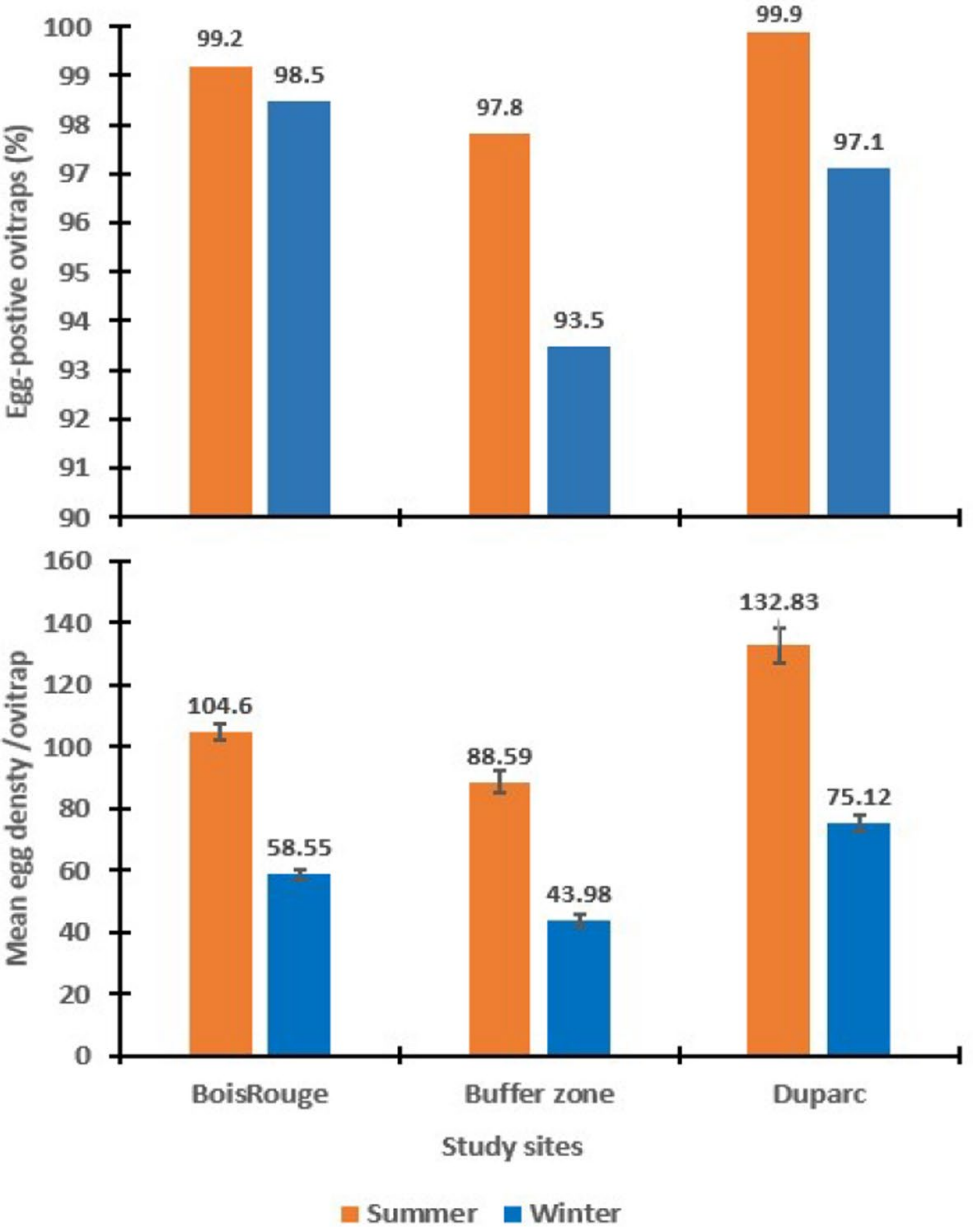
Mean proportion of egg-positive ovitraps (top panel) and mean numbers of *Ae*. *albopictus* eggs per ovitrap—week (low panel) at residences in study neighborhoods over the entire sampling period, split by season. The numbers on the bars indicate the mean values in different locations over the sampling period. The numbers of ovitraps used and in each study site are provided in Supplementary Table S2

When the data recorded at each site over the sampling period were allocated by season (Fig. 3), there is a marked seasonal difference in the frequency of egg-positive traps in Duparc (summer: 99.9%, winter: 97.1%, *χ*^*2*^ = 36.24, df = 2, *P* < 0.001), but not in Bois-Rouge (summer: 99.2%, winter: 98.5%, *χ*^*2*^ = 1.47, df = 2, *P* = 0.47). The seasonal difference was also pronounced in the buffer zone (summer: 97.8%, winter: 93.5%, *χ*^*2*^ = 12.60, df = 2, *P* = 0.002) (Fig. 3). Differences in the observed proportion of egg-positive traps between months or seasons were mainly related to variations in maximum temperature (*χ*^*2*^ = 9.54, df = 1, *P* = 0.002,) and temperature amplitude (*χ*^*2*^ = 12.14, df = 1, *P* < 0.001), but not in response to variations in rainfall (*P* = 0.50) and other climatic factors. As it would be unpractical to present all ovitraps observations over a long set of time points, because of the amount of space required, the data have been combined to show the average monthly values derived from original weekly records of eggs collected by ovitraps in each study site for each year (see supplemental file 1) and classified by season (Supplementary Table S2).

### Temporal variations of *Aedes* albopictus egg productivity throughout the year

Over 814,255 *Ae. albopictus* eggs (Total: 407,197 in Duparc, 347,138 in the Bois-Rouge, and 59,920 in the buffer zone) were recorded throughout the study period. There was a broad variation in the number of eggs per ovitrap per week in Duparc (range: 0–2124 eggs per ovitrap), Bois-Rouge (0–937), and the buffer zone (0–1135). The average egg densities per ovitraps varied among study sites (Mixed model analysis—fixed effect of site: *F*_2, 9534_ = 14.60, *P* < 0.001). Overall, multiple comparisons using Bonferroni adjustment showed that the difference in mean numbers of *Ae*. *albopictus* eggs per ovitrap was significantly higher in Duparc (weekly avg. = 99.47 ± 2.68 eggs/trap, *n* = 4166 observations) compared to that of Bois-Rouge (83.25 ± 2.31 eggs/trap, *n* = 4461 observations) (*F*_2, 9534_ = 7.94, *P* = 0.004). Ovitraps set in the buffer zone at the edge of the residential areas collected on average 65.84 ± 3.06 eggs per week (from 906 observations), which was marginally lower than the traps placed in the residential areas (Duparc and Bois-Rouge combined) (*F*_1, 8839_ = 3.89, *P* = *0.049*). The maximum number of eggs in a single ovitrap was observed in December 2013 in Duparc (*n* = 2124 eggs) and in March 2018 in Bois-Rouge (*n* = 937 eggs).

The distribution of the monthly mean number of eggs collected per ovitrap in each study site and year is given in Fig. 2. Data from all study sites showed seasonal dynamics of *Ae. albopictus* egg densities consisting of one single peak in density occasionally occurring as early as December and, more regularly February. Except in 2014, the average egg densities/ovitrap during the peak period tended to be higher in Duparc (≥ 200 eggs/trap) than in Bois-Rouge (≥ 150 eggs/trap) and the buffer zone (≥ 110 eggs/trap) (Fig. 2). The peak egg density was followed by a rapid decrease from early April onwards until September, after which there was a consistent rising trend from October to March, with occasional rebounds notably at the beginning of November. The same pattern was maintained across consecutive years from 2013 to 2018 in the urban as well as in the buffer zones. In this buffer zone, the greatest monthly number of *Ae. albopictus* eggs observed in a single ovitrap reached 1135 in February 2015. Finer statistical analyses using GLM showed significant variation in the number of eggs per trap between sampling dates (effect of month: *F*_10, 9483_ = 44.82, *P* < 0.001). When the data from each site were classified by year, significant inter-annual differences in the relative abundance of *Ae. albopictus* eggs/ovitrap emerged both in Bois-Rouge (Effect of year: *F*_4, 4411_ = 35.70, *P* < 0.001) and Duparc (*F*_5, 4411_ = 22.74, *P* < 0.001) but not in the buffer zone (*F*_2, 977_ = 2.51, *P* = 0.081). The difference in mean number of eggs between ovitraps set in the three sites varied inconsistently between year (area × year: *F*_5, 9483_ = 97.06, *P* < 0.001, Fig. 2). A striking feature of the monthly records is the consistent seasonal fluctuation pattern for *Ae. albopictus* eggs. Within a given study site (Fig. 3), there was statistically significant difference between egg densities recorded in the summer and the winter, in Duparc (*F*_1, 4567_ = 36.08, *P* < 0.001), Bois-Rouge (*F*_1, 4624=_ 269.52, *P* < 0.001) and in the Buffer zone (*F*_1, 946_ = 72.93, *P* < 0.001). Regardless of the season, the post-hoc analysis showed significantly higher egg abundance in Duparc than in Bois-Rouge and the buffer zone.

### Time series of the weather conditions during the study period considered for analysis

Figure 3 shows a time series of monthly precipitation and mean temperature data extracted from the Gillot Airport weather station for the considered study period, 2013–2018. Monthly average mean air temperatures remained rather constant every year (Fig. 3). During the study period, the daily mean temperature ranged from 21.7 °C to 27.6 °C. Temperatures usually have a typical rise from November to March, making January/February, on average, the hottest month of the year. After that, the mean monthly temperatures dropped steadily until June. During the winter, the months of June, July and August had the biggest deviation from the average, with a mean monthly temperature around 2 degrees below average across the studied sites. The succession of dry and rainy seasons in the study areas is visible in the precipitation graphs, with marked irregularity from one year to another (*F*_4, 10,094_ = 4451.36, *P* < 0.001). The months of July and August had the biggest variances in rainfall throughout the study period. The wettest months during the survey period were January, February, and March, during which the highest monthly rainfall ranged from 481.5 mm in January 2014 to 720.3 mm in January 2018 (Supplementary figure S1). The driest period with the lowest total rainfall of about 7.6 mm and 16.6 mm was recorded in June 2014 and August 2018, respectively. Regardless of the month, the total amount of precipitation was lowest in 2014 (95.1 ± 36.66 mm) and 2016 (93.04 ± 29.44 mm), while 2018 (178.42 ± 65.35 mm) was very rainy compared to other years*.*

### Relationship between Ae. albopictus egg-laying activity and spatiotemporal variation in climatic factors

To determine effects of weather characteristics on egg abundance, we used generalized linear mixed effects models (GLMMs) with a negative binomial link to evaluate the effect of meteorological variables on the egg abundance while including study site as random effect. Whereas the weekly variation in egg density/per ovitrap was positively related to rainfall, mean and maximum temperature and minimum relative humidity (Table 1), negative but significant relationships were found been ovitrap productivity and other weather parameters considered (*P* < 0.001 in all cases).

**Table 1:** Summary of meteorological parameters affecting the variation in the monthly mean counts of *Aedes albopictus* eggs according to the selected Generalized linear mixed model (GLMM)

Estimates of Fixed Effects^a^							
Parameter	Estimate	Std. Error	*df*	*t*	Sig.	95% confidence interval	
						Lower bound	Upper bound
Intercept	− 85.87	82.79	6665.54	− 1.03	0.300	− 248.18	76.43
Rainfall	0.05	0.012	9479.96	4.19	< 0.001	0.028	0.078
Tmax	50.99	32.89	9531.59	1.55	0.121	− 13.48	115.47
Tmin	− 197.00	28.03	9527.07	− 7.02	< 0.001	− 251.95	− 142.04
Tmean	158.81	34.08	9531.65	4.65	< 0.001	91.99	225.62
TAmplitude	− 97.60	26.276	9527.99	− 3.71	< 0.001	− 149.10	− 46.09
Rhmin	1.51	0.27	9532.33	5.47	< 0.001	0.97	2.057
Rhmax	− 2.69	0.68	9533.53	− 3.942	< 0.001	− 4.038	− 1.35
Rhmean	− 1.31	0.87	9532.46	− 1.502	0.133	− 3.035	0.40
FFM	− 5.09	3.23	9532.61	− 1.574	0.115	− 11.43	1.24
INST	− 0.007	0.001	9532.73	− 5.791	< 0.001	− 0.009	− 0.0047
GLOT	− 0.00042	0.00019	9531.63	− 2.240	0.025	− 0.0007	− 5.31E−5

^a^Dependent Variable: egg count; Rainfall: Average monthly precipitation; *Tmean* monthly mean of daily average temperature, *Tmax* monthly mean of daily maximum temperature, *Tmin* monthly mean of daily minimum temperature, *TAmplitude* monthly mean of daily thermal amplitude, *Rhmean* monthly mean of daily average relative humidity, *Rhmin* monthly mean of daily minimum relative humidity, *Rhmax* monthly mean of daily maximum relative humidity, *FFM* monthly mean of daily average wind speed, *INST* cumulative total insolation times per month, *GLOT* cumulative daily global solar radiation per month

Among these, monthly mean maximum temperatures, and monthly mean temperature both showed significant association with monthly variations in mean egg densities (*P* < 0.001 in all cases, Table 1), with the partial correlation values at 0.22, 0.21 and 0.22, respectively. This suggest that either a monthly rise in mean temperature or a higher maximum and lower minimum temperature were positively associated with an increase in *Ae. albopictus* egg-laying activity in the field. Positive association was also found between egg densities and rainfall (*β* = 0.05, t = 4.19, *P* < 0.001) (Table 1), with higher precipitation leading to increases in egg densities of 17%. Monthly averages of daily wind velocity, total daily sunshine duration, and cumulative daily global solar radiation also contributed significantly in the monthly variation of egg density, although overall, the number of eggs showed a negative correlation with monthly average of daily wind speed (*r* = -0.16, *P* < 0.001) and the monthly degree of insolation (*r* = -0.11, *P* < 0.001).

### Spatiotemporal variation in the distribution of eggs across the study areas

**Fig. 4:**
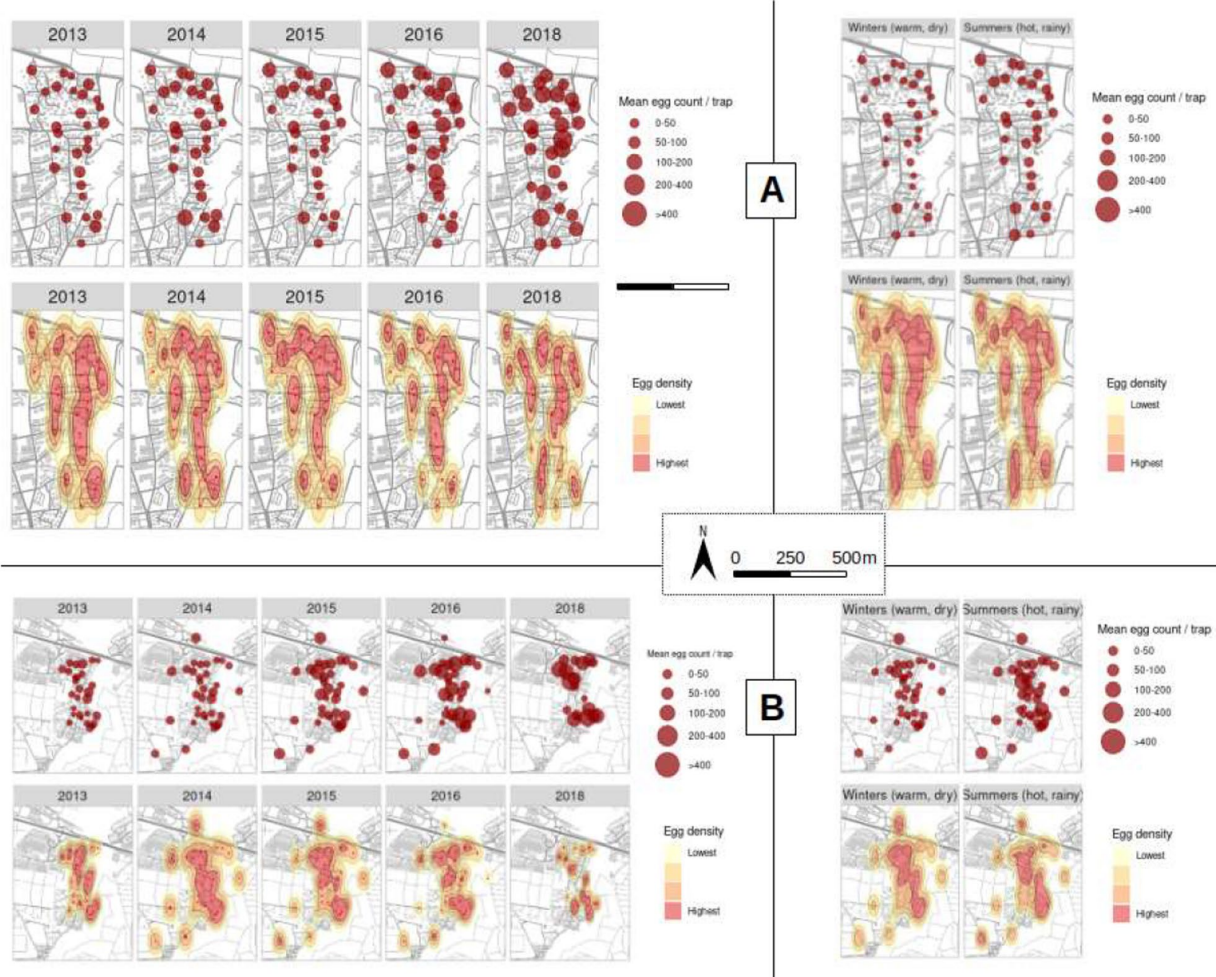
Spatiotemporal variation of the relative abundance of *Aedes albopictus* eggs in ovitraps in Bois-Rouge (**A**) and Duparc (**B**). The spatial representation shows the variability of ovitrap productivity defined as monthly total egg abundance in all ovitraps for each year and season at the three study sites: low suitability areas were defined with egg density < 100 eggs/ovitrap, intermediate suitability with 100–200 eggs/ovitrap, and high suitability when egg density is > 200 eggs/ovitrap

**Fig. 5:**
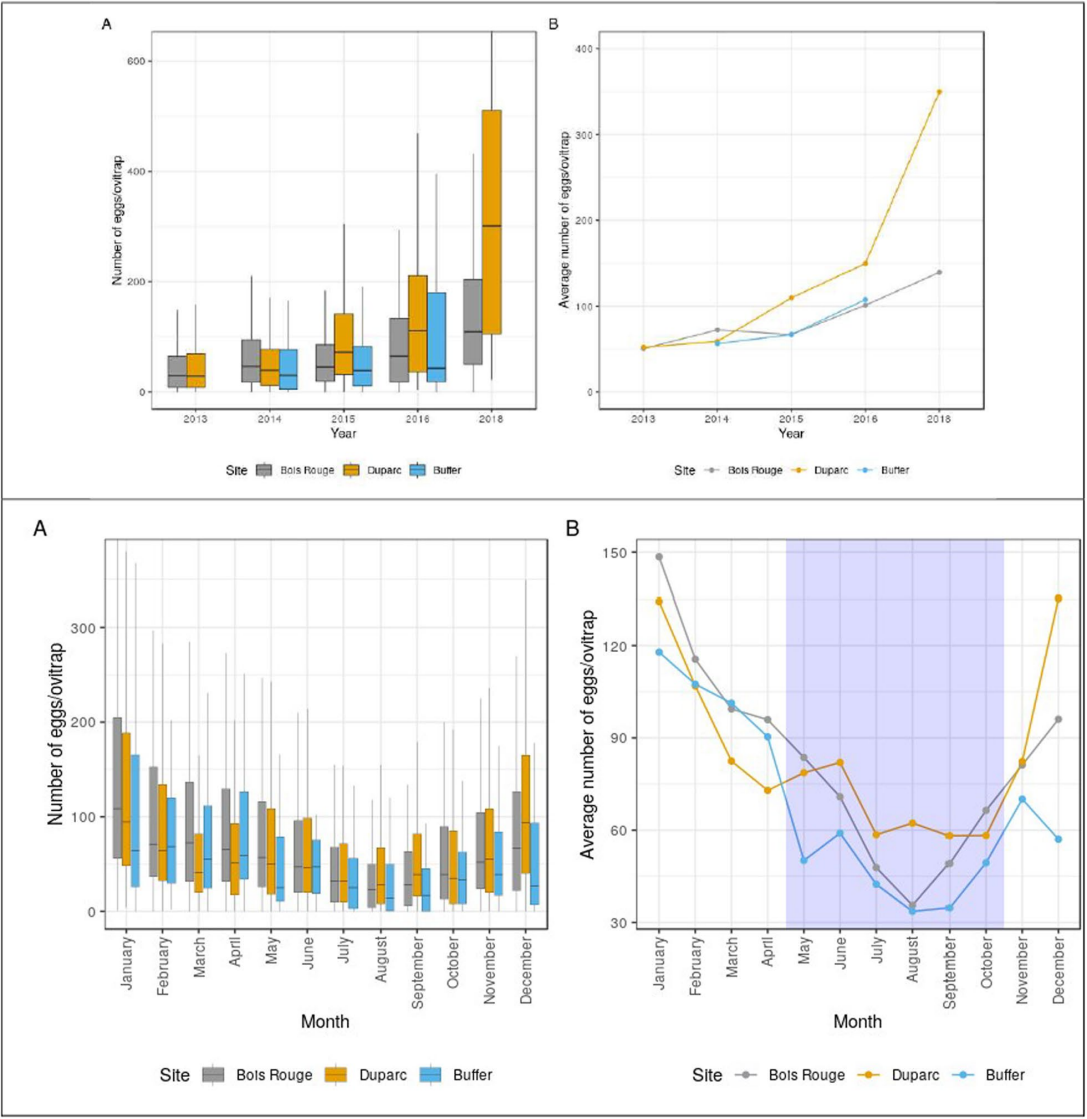
Comparison of temporal dynamics of recorded *Ae. albopictus* egg densities in residential (Bois-Rouge and Duparc) and non-residential buffer zones.The first panel shows the distribution of the yearly total (**A**) and yearly average (**B**) egg density per ovitraps in the different years, whereas the second panel show the monthly total (**A**) and monthly average (**B**) egg density per ovitraps at the surveyed study areas

Suitability maps (Fig. 4, appendix 3) show that in the three study areas, there was a spatial gradient of suitability with clearly localized areas of high egg densities per ovitrap. The study area in Bois-Rouge shows patchiness, where there is geographically concentrated oviposition activity in the northeastern compared to the rest of the mapped area with small-scattered patches of low egg density per ovitrap. In Duparc, Fig. 4 also shows areas of continued space–time clustering in *Ae. albopictus* oviposition, with higher density for *Ae. albopictus* eggs produced from ovitraps placed at the northwestern and southeastern parts facing the ravine and the agricultural fields. The central area of Duparc showed intermediate to high *Ae. albopictus* egg counts in ovitraps throughout the study period. Close examination of the yearly maps (Fig. 4) indicate that the incidence of high egg densities increases as the rainy season progresses (from November to April) and then starts to decrease in the winter, from May–June in all sites. Although the hotspot areas for *Ae. albopictus* oviposition in specific parts across the studied sites remain stable throughout, the frequency of ovitraps with > 200 eggs increases from year to year (Figs. 4 and 5) and rose from 3.0% in 2013 to over 25.7% in 2018 in Bois-Rouge, and from 4.6% in 2013 to 21.3% in 2018 in Duparc. Besides the time-varying effect of weather conditions, this increase in high egg density is probably linked to changes in environmental conditions. Figure 5 compares the distribution of egg density in the residential vs non-residential area. Localized clusters associated with high egg densities were concentrated in residential areas, namely to the northwest of Duparc and in the northeast of Bois-Rouge.

## Discussion

This study used ovitraps to provide important insights into the spatial and temporal dynamics of *Ae. albopictus* oviposition activity in specific urban settings chosen for the future release of sterile males as part of a pilot program to suppress *Ae*. *albopictus* populations. Repeated observations over multiple years confirmed that *Ae*. *albopictus* is present year-round in the study area, with consistent interannual variation in oviposition activity. Analysis weekly and monthly time series data on the incidence of egg traps and the number of eggs over six years, from 2013 to 2018, showed spatial and temporal trends in female egg-laying activity and the extent of its correlation with climatic variables. The superimposed trends in average *Ae. albopictus* egg densities in Duparc and Bois-Rouge exhibited the same seasonality pattern, albeit with different magnitudes from year to year, indicating the absence of a regular periodicity in this seasonality. Overall, the oviposition activity of the wild *Ae. albopictus* population monitored through egg densities in ovitraps typically declined during the cool and dry winter season, with the lowest average egg densities consistently observed from May to July, and occasionally during the second half of the winter season, in August and September. Subsequently, mosquito breeding gradually increased from November to March, peaking in January/February regardless of the surveyed periods. Collectively, the results from ovitraps monitoring over multiple years demonstrated that the presence of wild *Ae. albopictus*, along with their physiological egg-laying activity, is continuous in the two study sites.

In general, the results demonstrated that the presence of *Ae. albopictus* females, as well as their physiological egg-laying activity, remained high throughout the year, with the proportion of egg-positive traps often exceeding 80% or even 95%. As this pattern was consistent across the study site throughout the study period, it can be concluded that this positivity index is probably not sensitive enough to provide a good indicator of the effectiveness of a vector control method applied for *Ae. albopictus* control. It should be noted beforehand that the use of ovitraps for egg sampling may not represent a proxy of *Ae. albopictus* adult population size in the study area, nor can it capture the exact proportion of all the niches available to egg-laying females at the time samples were taken [38, 39]. Although rarely explored for field populations of *Ae. albopictus*, however, density-dependent effects on reproduction are likely to occur. Several biotic factors such as fluctuation of host availability and man-biting adult mosquito populations, reproductive rate or the natural growth of the population, female survival rate, and any possible behavioral interference among feeding or ovipositing females can influence the dynamics of the wild *Aedes* sp. population. All other things being equal, we can deduce that the marked spatial and seasonal patterns of egg abundance in ovitraps would be influenced by a combination of population size and adult activity at the time of sampling. Besides, abiotic factors (e.g. climatic and environmental factors) may also contribute to the total variability concerning female oviposition activity [40, 41], survival and distribution [42–44]*.*

### Influence of climatic factors on *Ae. albopictus* oviposition activity

Because an annual seasonality is observed in egg count data from ovitrap, it is appropriate to consider the relative importance of key meteorological factors affecting the temporal oviposition dynamics of *Ae. albopictus* in our study sites. Owing to the geographic proximity of the two sites under investigation, it is postulated that climatic variables, such as temperature and rainfall, have a uniform impact on them. Nevertheless, previous studies conducted in different contexts have proposed a correlation between climate and the variation in population dynamics of *Ae. albopictus* [45]. A review of the literature revealed that mosquito activities are more favorable within a temperature range of 15 °C to 28 °C [46]. The average monthly temperatures (20–25 °C) and humidity levels (60–70%) observed during winter in the study areas still fall within the suitable ranges for the development and reproduction of *Ae. albopictus*. Consistent with earlier research by Delatte et al. [47], it is anticipated that winter temperatures offer optimal conditions for the growth of aquatic phases and the survival of *Ae. albopictus* in Reunion Island. In the absence of rainfall for several months during the austral winter, consistently favorable temperatures and humidity levels are likely the primary factors facilitating continuous oviposition activity of wild *Ae. albopictus*. However, it is important to consider other environmental factors, in addition to temperature and relative humidity, to accurately predict the dynamics of *Ae. albopictus* oviposition activity in the study areas. The decrease in oviposition activity over 5 months (May to September) may indicate that reproduction is regulated by density-dependent factors, such as reduced adult longevity and activity [48, 49], as well as the scarcity of larval habitats [50].

In contrast with the winter, the exponential increase in the size of egg batches found in ovitraps during the hot and rainy summer is related to continuously high temperatures, high rainfall, and availability of rain-fed outdoor containers. The inter-annual variability of rainfall is reflected in a corresponding variability in the abundance of the population of *Ae. albopictus* each year (Fig. 4). It is generally assumed that superabundant precipitations may result in flushing breeding habitats, which negatively influences the development of the larvae to pupae, the emergence of new adults, and the copulation and host-seeking behavior of the adults [51]. The frequent occurrences of rainstorms in January–March and heavy rainfall in April–May probably led to most mosquito habitats being inundated and refilling those habitats that were beginning to stagnate during the drier months. Regardless of their flushing effect, the year-round presence of abundant *Ae. albopictus* eggs indicate that the amount of rainfall (200–400 mm during the summer months), as well as their period of occurrence in our study sites, was not a limiting factor to the reproduction activity and the development of the species. During the hot and humid season, average temperatures were generally greater than 27 °C, with average minima systematically in the range 23–25 °C. In addition, strong gusts of wind are frequently observed at the beginning of each year in the north of Reunion. These strong wind events may considerably dry out the ambient air that causes a potential deterrent effect on mosquito flight patterns and behavior and interfere with the mosquito's ability to fly and thus be unable to move to locate potential hosts, food source and as well as breeding sites [52], yet trap positivity rates remained generally between 80 and 90% and egg densities per ovitrap remained high.

Inter-annual variability introduces a level of complexity that makes analysis less straightforward. Challenges arise when interpreting counts of eggs specific to certain areas, especially when these counts undergo rapid changes in the short term and when there are areas with high egg concentrations. The presence of hotspots throughout the year, even for the winter season, may be attributed to the selective behavior of mosquito females, which seems to demonstrate a preference for certain environments over others. In agreement with previous findings in Mauritius [53], this study reveals that the highest number of eggs were produced in residential areas where the abundance of *Ae. albopictus* could be due to increased availability of both artificial and natural breeding sites. However, the primary sources for the high populations in residential areas are man-made containers such as pools, tires, buckets, eaves, troughs, and similar receptacles found on private properties in the surrounding neighborhoods [22, 23]. Additionally, there was a relatively high occurrence of *Ae. albopictus* eggs near the ravine and agricultural fields bordering the residential areas. Various environmental factors also influence the activity of adult *Ae. albopictus* in natural areas. The seasonal and regular spatio-temporal distribution of *Ae. albopictus* populations in all areas examined, with a greater increase in egg-laying during the summer compared to the winter season, align with those reported by other studies in different contexts [45, 54]. Similar studies focusing on *Ae. aegypti* have shown that fluctuations in temperature and rainfall or irregular distribution of breeding sites, caused both temporal and spatial variations in population abundance [46, 55].

Seasonal population fluctuations are common in the majority of *Aedes* sp. mosquito species [30, 51]. This is because an adult population with a diverse distribution in physiological age over an extended period will inevitably undergo cyclic changes. It is reasonable to hypothesize that there is a complex and subtle relationship between weather parameters and the seasonality of egg-laying activity across a broad geographical range. To gain a better understanding of this phenomenon, Tran et al. [5] utilized data from a comprehensive and long-term study on the yearly fluctuations in *Ae. albopictus* populations in Reunion Island, to investigate potential responses to different weather conditions. The modeling approach generated various patterns of seasonal fluctuation and revealed that the low densities of *Ae. albopictus* during the winter season likely results from the constraints imposed by specific environmental characteristics and the adaptive mechanisms developed by mosquitoes in urban environments. This analysis specifically takes into consideration the uniqueness of different conditions encountered in Reunion Island.

### Spatial variation in the distribution of egg-laying activity

The residential areas, which are inhabited by dense human populations, exhibited higher densities of *Ae. albopictus* eggs in the ovitraps as compared to the sparsely populated surrounding natural areas and farmland. The allocation of eggs among oviposition sites may be subject to the influence of specific site conditions, resulting in "preferred" sites receiving a greater number of eggs while "non-preferred" sites receive more limited contributions. However, it is important to note that the monitoring in the buffer zone was conducted within limited timeframes (22 months, from April 2014 to January 2016), thus any conclusions drawn should be cautiously interpreted in light of the constrained observation time. Nevertheless, landscape features such as human population density and habitat alteration play an important role in the oviposition behavior and influence the distribution and population spread of *Aedes* vectors [56]. Anthropogenic activities in urban areas can create artificial oviposition sites that benefit container-breeding mosquito species [57], including *Ae. albopictus*. Recent studies have shown that *Aedes* species, including *Aedes aegypti* and *Ae. albopictus*, exhibit breeding preferences for specific habitats in the urban environment, such as water storage containers [58]. The spatial distribution of these habitats also affects oviposition, with clustered patches attracting more ovipositing mosquitoes than dispersed patches [59]. Additionally, the occurrence of low egg-laying activity could be triggered by the presence of certain landscape features, such as green areas and pavements, known to negatively influence mosquito abundance, while exposed soil and open land positively influence their presence [60]. Further studies comparing an array of different urban settings would be essential to fully understand how landscape heterogeneity and habitat characteristics affect mosquito abundance and dynamics on a spatial and temporal scale. The observed spatial variations in the oviposition activity with localized hotspot may also be driven not only by variation in adult population and activity—which are themselves likely related to climate, but also by a complex interplay of landscape characteristics, availability of blood meals and suitable breeding sites. Although it is assumed above that climatic conditions are crucial in increasing or decreasing oviposition activity, determining whether environmental factors play a causal role in oviposition activity will be essential for designing effective SIT intervention.

This report is restricted only to longitudinal monitoring of egg-laying activity using ovitraps. It is limited by the completeness of the information on adult population dynamics. As imperfect as the ovitrap monitoring systems might be, there is no obvious reason why the seasonal peaks detected here would be pure artifacts. Our results and conclusions are based on the relative seasonal variations in egg abundance and the peak timing, not on the absolute magnitude of the peak. Inherent to our analysis is the assumption that while the magnitude of *Ae. albopictus* population dynamics might be underestimated using ovitrap [39], the seasonal peaks are more likely due to the intrinsic dynamics of the activity of females than to systematic biases in the monitoring systems. The consistency of the observed trends in several years across the three study sites suggests that this assumption is reasonable. Knowledge of the factors determining the reported seasonality in *Ae. albopictus* egg abundance is crucial for developing appropriate prevention and control strategies, especially given the potential for targeted SIT strategies. Special attention should be paid to the optimal time of application: indeed, if insecticide treatments are usually started and repeated during the year when mosquito abundance is high in the field [61], modeling studies suggest that applying SIT when the population density is low may be more efficient [62]. Recent modeling approaches also addressed other conditions of use such as the release frequencies and the minimal percentage of the targeted mosquito population affected by the treatment [63, 64], or the impact of residual female contamination or residual fertility on the effectiveness of SIT [65]. The present study also highlights the importance of considering the spatial structure of the urban landscape when implementing mosquito control strategies.

## Conclusions

Our efforts to uncover the dynamic patterns of *Ae. albopictus* population constitutes a first step towards generating hypotheses about how multiple factors interact to produce the complex dynamics exhibited by this species. Our finding confirmed that climatic conditions with consistently high temperatures, regular rainfall, and high humidity conditions create ideal conditions for *Ae. albopictus* survival, reproduction and proliferation in Reunion Island. The findings revealed the dynamics of *Ae. albopictus* egg-laying activity with considerable variations throughout the study period. The approach taken and the results obtained deepen our knowledge of the seasonal dynamics of *Ae. albopictus* populations and the interplay of biotic and abiotic factors in the studied urban settings. In this study, only part of the ecological factors (climatic variables and gross habitat) were examined as data on other factors (e.g., land cover, housing conditions, and personal protection behavior) was unavailable. Our results suggest, however, that multiple and varied site-specific drivers might interact with seasonal factors to determine the ecology of *Ae. albopictus*. Further studies are needed to investigate more broadly the combination of weather, and environmental factors that could account for these spatial and temporal variations in the *Ae. albopictus* population dynamics and their response to SIT-based vector control. Early modeling studies have suggested that rainfall and temperature play major roles in the effectiveness of SIT vector control strategies [63, 66]. Such forecasts are of central importance for prioritizing SIT interventions or forecasting outcomes for different areas. Taking into account previous models and the results of the present study, the mid-winter period (mid-July to September) represents a crucial time for the implementation of SIT, since it is precisely when densities of wild mosquito population are lowest that the sterile male release technique is most likely to have an impact on the reproductive dynamics of wild populations. In addition, the relatively high population density observed in this study suggests an important role for the removal of breeding habitats as a complementary approach to reduce vector population prior to SIT application. Lastly, the best use of these tools would be to target the sterile male release to area where human population is directly exposed—in the residential areas, while accounting for the focal hotspots in the targeted neighborhoods and the possible immigration *Ae. albopictus* females from the surrounding natural and agricultural lands. The primary application of this study will be its use to test the efficacy of SIT in reducing the fecundity and fertility of *Ae. albopictus* in the characterized urban areas.

## Supplementary Information

Supplementary Material 1.

## Data Availability

The raw entomological data produced during this study and described in this manuscript are available online: 10.15468/q4tq5q [67]. Additional information generated during data analysis are included in this published article and its supplementary information files.

## Abbreviations

SIT: Sterile insect technique
Tmean: Monthly mean of daily average temperature
Tmax: Monthly mean of daily maximum temperature
Tmin: Monthly mean of daily minimum temperature
TAmplitude: Monthly mean of daily thermal amplitude
Rhmean: Monthly mean of daily average relative humidity
Rhmin: Monthly mean of daily minimum relative humidity
Rhmax: Monthly mean of daily maximum relative humidity
FFM: Monthly mean of daily average wind speed
INST: Cumulative total insolation times per month
GLOT: Cumulative daily global solar radiation per month
