# Supplementary material for: Spatial and temporal characterization of Aedes albopictus oviposition activity in candidate urban settings for sterile insect technique testing in La Reunion Island
Source: Infect Dis Poverty. 2024 Oct 25;13:78. doi: 10.1186/s40249-024-01250-z (PMC11515404; doi:10.1186/s40249-024-01250-z)
Supplement: Supplementary file 1 — Supplementary Material 1. [file 40249_2024_1250_MOESM1_ESM.pdf]

Supplementary materials

Supplementary table S1. Monthly averages of meteorological variables calculated using daily records during the study period. The differences in monthly averages between years is shown in Table 2

Parameters	Mean	SEM	Minimum	Maximum
Rainfall (mm)	108.14	1.320	7.6	738.4
Tmean	24.43	0.018	21.7	27.5
Tmin	20.90	0.019	17.6	24.3
Tmax	27.98	0.017	25.4	31.2
TAmp	7.08	0.004	6.0	8.3
Rhmean	70.04	0.033	64	78
Rhmin	39.42	0.06	23	55
Rhmax	92.73	0.022	89	98
FFM	5.510	0.006	3.8	6.7
INST	13,291.68	12.691	9,669	16,731
GLOT	58,035.81	97.660	37 ,134	79,784

Tmean: monthly mean of daily average temperature (° C). Tmax: monthly mean of daily maximum temperature (°C). Tmin: monthly mean of daily minimum temperature (°C). TAmp: monthly mean of daily thermal amplitude (°C). Rhmean: monthly mean of daily average relative humidity (%); Rhmin: monthly mean of daily minimun relative humidity (%). Rhmax: monthly mean of daily maximum relative humidity (%). FFM: monthly mean of daily average wind speed (m/s). INST: cumulative total insolation times per month (in mn). GLOT: cumulative daily global solar radiation per month (in J/cm^2) SEM: Standard Error of Mean; mm: millimeter; °C: degree Celsius; %: percent.

Supplementary table S2. Seasonal fluctuation of the number (%) of egg-positive ovitraps and mean (+ SD) number of *Ae. albopictus* across study sites.

		Duparc	Bois-Rouge	Buffer zone
	Nb of observations	4569	4626	948
**Summer**	Nb positive (%)	1631_a (86.3)	1851_a (95.0)	440_a (94.2)
	Mean (SE) eggs density	133.00 (3.72) $_a$	105.45 (2.50) $_a$	90.60 (5.65) $_a$
**Winter**	Nb positive (%)	2459_b (91.8)	2557_a (95.5)	425_b (88.4)
	Mean (SE) eggs density	77.38 (2.07) $_b$	59.45 (1.41) $_b$	47.19 (2.76) $_b$

The value with different letter indicate significant difference (Independent T-test accounting for unbalance sample sizes) in the two dependent variables between seasons. Except in Bois-Rouge where number of egg-positive traps did not vary with season, there was statistical evidence of significant seasonal differences ($P < 0.001$) in the proportion in all study site. There was seasonal fluctuation of ovitrap productivity in each study site, with lower egg densities were observed during winter compared with that recorded in the summer. Moreover, the results suggest that ovitraps placed in the buffer zone had lower mean egg densities than those placed in urbans areas , but the difference was significant when comparing Duparc vs. Buffer zone, $T = - 3.61$, $df = 4953$, $p < 0.001$, whereas the difference in ovitrap productivity between Bois-Rouge et the Buffer zone was not statistically (T-test = 1.70, $df = 5727$, $p = 0.089$).

Supplementary figure S1

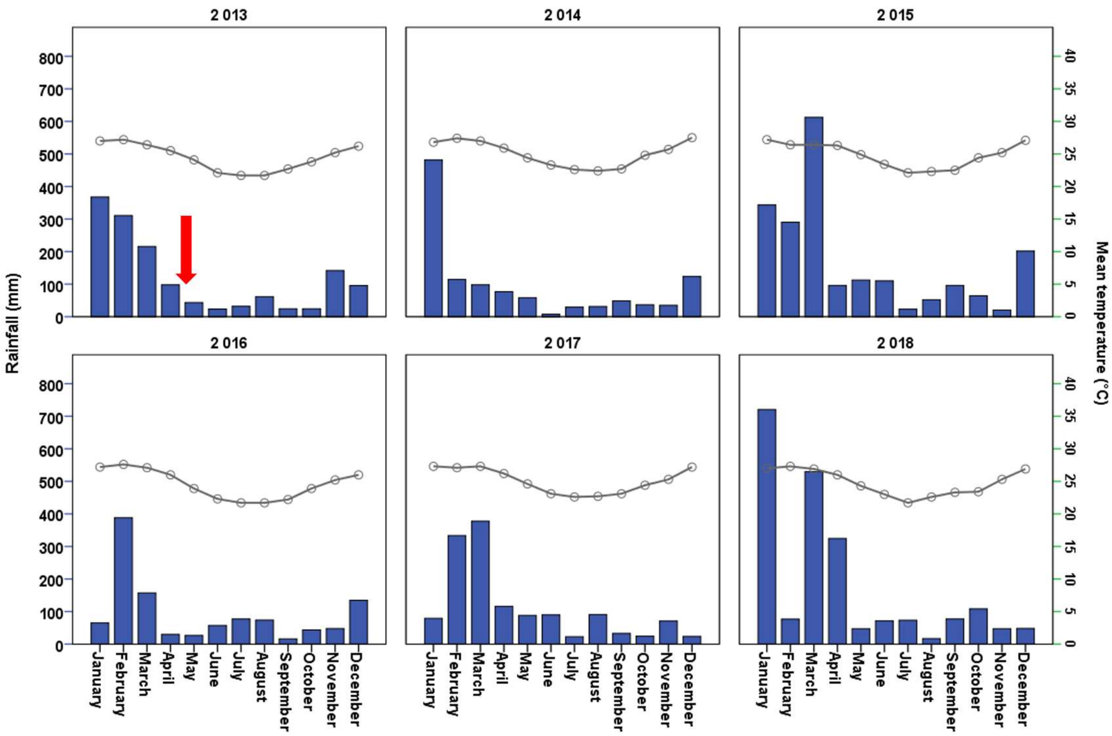

Supplementary Figure S1. Monthly mean temperature (dashed lines, in °C) and monthly total precipitation (bars, in millimeter, mm) in the municipality of Sainte Marie, from May 2013 to December 2018. The arrow indicates the start of the sampling period.
